# Emotional valence and arousal affect reading in an interactive way: Neuroimaging evidence for an approach-withdrawal framework

**DOI:** 10.1016/j.neuropsychologia.2014.01.002

**Published:** 2014-04

**Authors:** Francesca M.M. Citron, Marcus A. Gray, Hugo D. Critchley, Brendan S. Weekes, Evelyn C. Ferstl

**Affiliations:** a Cluster of Excellence “Languages of Emotion”, Freie Universität Berlin, Habelschwerdter Allee 45, D-14195 Berlin, Germany; b Centre for Advanced Imaging, The University of Queensland, Australia; c Psychiatry, Brighton & Sussex Medical School, Universities of Brighton and Sussex, UK; d Sussex Partnership NHS Foundation Trust, Brighton, UK; e Sackler Centre for Consciousness Science, University of Sussex, UK; f Laboratory for Communication Science, The University of Hong Kong, China; g Center for Cognitive Science, Albert-Ludwigs-Universität Freiburg, Germany

**Keywords:** Valence, Arousal, Approach, Withdrawal, Emotional words, fMRI

## Abstract

A growing body of literature shows that the emotional content of verbal material affects reading, wherein emotional words are given processing priority compared to neutral words. Human emotions can be conceptualised within a two-dimensional model comprised of emotional valence and arousal (intensity). These variables are at least in part distinct, but recent studies report interactive effects during implicit emotion processing and relate these to stimulus-evoked approach-withdrawal tendencies.

The aim of the present study was to explore how valence and arousal interact at the neural level, during implicit emotion word processing. The emotional attributes of written word stimuli were orthogonally manipulated based on behavioural ratings from a corpus of emotion words. Stimuli were presented during an fMRI experiment while 16 participants performed a lexical decision task, which did not require explicit evaluation of a word′s emotional content.

Results showed greater neural activation within right insular cortex in response to stimuli evoking conflicting approach-withdrawal tendencies (i.e., positive high-arousal and negative low-arousal words) compared to stimuli evoking congruent approach vs. withdrawal tendencies (i.e., positive low-arousal and negative high-arousal words). Further, a significant cluster of activation in the left extra-striate cortex was found in response to emotional than neutral words, suggesting enhanced perceptual processing of emotionally salient stimuli.

These findings support an interactive two-dimensional approach to the study of emotion word recognition and suggest that the integration of valence and arousal dimensions recruits a brain region associated with interoception, emotional awareness and sympathetic functions.

## Highlights

• Emotional valence and arousal affect reading interactively.
• Positive high-arousal and negative low-arousal words evoke conflicting reactions.
• Enhanced right insula activation was found in response to conflicting stimuli.
• Insula integrates viscero-sensory and cognitive/evaluative information.
• Enhanced extra-striate cortex activation was found for emotional than neutral words.

## 1 Introduction

A growing body of literature shows that the emotional content of verbal material affects reading (Citron, 2012; Kissler, Assadollahi, & Herbert, 2006). In particular, emotionally-laden words are processed faster and more accurately than neutral words (Kousta, Vinson, & Vigliocco, 2009; Larsen, Mercer, & Balota, 2006), they elicit larger amplitudes of electrophysiological components associated with emotion processing (Kissler, Herbert, Peyk, & Junghofer, 2007), and they yield enhanced BOLD responses in limbic brain regions (Kuchinke et al. 2005).

Several theoretical models of emotion have been proposed, including amongst others, models which propose a small number of universal underlying emotional states, i.e., discrete emotions such as joy, fear, etc. (see Levenson, 2011 for a review), appraisal models, which suggest that specific emotions are importantly influenced by appraisal processes which integrate the situational context of an event (see Ellsworth & Scherer, 2003), and dimensional models, which may be particularly useful for investigating the emotional processing of language.

Dimensional models suggest that emotion is best understood as occurring within a dimensional space, most commonly a two-dimensional space spanning valence and arousal. Emotional valence describes the extent to which an emotion is positive or negative, whereas arousal refers to its intensity, i.e., the strength of the associated emotional state (Feldman Barrett & Russell, 1999; Lang, Bradley, & Cuthbert, 1997; Russell, 2003). These models typically assume valence and arousal to be at least in part distinct dimensions (Feldman Barrett & Russell, 1999; Reisenzein, 1994). However, behavioural ratings of emotion word stimuli show that highly positive and highly negative stimuli tend to be more arousing (Bradley & Lang, 1999) and negative stimuli are generally rated higher in arousal than positive stimuli (e.g., Citron, Weekes, & Ferstl, 2012).

Support for a distinction between these two dimensions comes from neuroimaging studies that demonstrate dissociable cortical representations during processing of odours, tastes, and written words. Specifically, the orbitofrontal and ventral anterior cingulate cortices respond more to valence, whereas the amygdala and anterior insular cortex respond more to arousal (Colibazzi et al., 2010; Lewis, Critchley, Rotshtein, & Dolan, 2007; Posner et al., 2009; Small et al., 2003; Winston, Gottfried, Kilner, & Dolan, 2005).

Despite this evidence, some empirical work shows that valence and arousal affect processing of emotional stimuli in an interactive way (Robinson, Storbeck, Meier, & Kirkeby, 2004). The authors propose a model according to which stimuli with negative valence (e.g., bitter taste) or with high arousal (e.g., a loud noise) elicit a withdrawal tendency and corresponding mental set, because they represent a possible threat; in contrast, stimuli with positive valence (e.g., sweets) or with low arousal (e.g., a newsletter) elicit an approach tendency because they are perceived as safe. According to this account, these two tendencies are initiated independently at a pre-attentive level and subsequently integrated in order to evaluate the stimulus for further action (Robinson et al., 2004). Thus, positive low-arousal and negative high-arousal stimuli will be easier to process because they elicit congruent tendencies (approach and withdrawal, respectively), whereas positive high-arousal and negative low-arousal stimuli will be more difficult to process because they elicit conflicting approach-withdrawal tendencies. According to this model, these opposite tendencies are integrated at an implicit processing level, before explicit stimulus evaluation.

In a series of experiments, Robinson et al. (2004) asked participants to judge the emotional valence (positive vs. negative) of pictures as well as written words. The results showed consistent interactive effects of valence and arousal, whereby reaction times (RTs) were faster for stimuli eliciting congruent approach or withdrawal tendencies compared to stimuli eliciting conflicting tendencies (Robinson et al., 2004). In these studies, participants were asked to explicitly evaluate the emotional connotation of the stimuli. Thus, it is difficult to tease apart whether the interactive effects of valence and arousal are caused by truly automatic processes, i.e., implicit integration of approach-withdrawal tendencies, or instead by intentional stimulus evaluation as well as strategic processes.

To this end, Eder and Rothermund (2010) devised a task to assess the emotional evaluations of pictorial stimuli indirectly and observed the same interaction reported by Robinson et al. (2004). Further support for implicit interactive effects of valence and arousal during reading of emotionally-laden words comes from studies using a lexical decision task (LDT), i.e., decide whether a letter string is a real word or a not. This task allows assessment of the implicit processing of a word′s emotional connotation (Bayer, Sommer, & Schacht, 2012; Citron, Weekes, & Ferstl, under review; Hofmann, Kuchinke, Tamm, Võ, & Jacobs, 2009; Larsen, Mercer, Balota, & Strube, 2008). Slower LD latencies are reported for words eliciting conflicting approach-withdrawal tendencies compared to words eliciting congruent tendencies.

Neural evidence for interactive effects of valence and arousal comes from studies showing modulation of the amplitude of emotion-related event-related potential (ERP) components during the implicit processing of emotional pictures (Feng et al., 2012) as well as words (Citron, Weekes, & Ferstl, 2013; Hofmann et al., 2009) (but see Bayer et al., 2012 for distinct ERP effects of the two emotional dimensions).

The aim of the present study is to test for interactive effects of valence and arousal on regional neural activity, in order to identify which brain regions are responsible for the implicit integration of approach-withdrawal tendencies during reading of emotionally-laden words. This is the first hemodynamic neuroimaging study to explore the interaction rather than the dissociation of emotional dimensions. In fact, previous functional magnetic resonance imaging (fMRI) studies have tested for the dissociation of brain activation between valence and arousal dimensions and employed either tasks requiring explicit and deep processing of a word′s emotional content (Colibazzi et al., 2010; Posner et al., 2009) or self-referential processing, which tends to evoke a bias toward “yes” responses to positively valenced words, which possibly enhances the processing of these trials (Lewis et al., 2007). Such studies support the multidimensional account of emotion processing, but do not speak to the interrelationship between valence and arousal during implicit emotion processing in reading.

Typically, the reading of emotionally-laden words in studies requiring implicit processing of their emotional content evokes activity within a set of brain regions that include the amygdala (Herbert et al., 2009; Kensinger & Schacter, 2006; Lewis et al., 2007; Straube, Sauer, & Miltner, 2011), the anterior cingulate cortex (ACC; Kuchinke et al., 2005; Lewis et al., 2007), the insula (Lewis et al., 2007; Straube et al., 2011), the prefrontal cortex (PFC; Compton et al., 2003; Kuchinke et al., 2005; Straube et al., 2011), more specifically the orbito-frontal cortex (OFC; Kuchinke et al., 2005; Lewis et al., 2007), the hippocampus, the parahippocampal gyrus (Kuchinke et al., 2005) and extra-striate cortical areas (Compton et al., 2003; Herbert et al., 2009).

In an event-related fMRI design, we presented participants with written positive and negative words, high or low in arousal, and neutral words. Stimuli were intermixed with non-words and participants performed a LDT, thus evoking implicit emotion processing. According to Robinson et al.′s model and the extant supportive empirical evidence, we predicted slower LD latencies, lower accuracy and enhanced BOLD signal response for words eliciting conflicting approach-withdrawal tendencies (i.e., positive high-arousal and negative low-arousal words) compared to words eliciting congruent tendencies (i.e., positive low-arousal and negative high-arousal words). More specifically, we expected enhanced BOLD responses in the insula and/or ACC. In fact, the former sub-serves affective/interoceptive awareness, i.e., integration of bodily sensations and cognitive, evaluative processes (Brooks, Zambreanu, Godinez, Craig, & Tracey, 2005; Craig, 2009; Critchley, Wiens, Rotshtein, Ohman, & Dolan, 2004), whereas the latter is associated with error detection (Botvinick, Nystrom, Fissell, Cater, & Cohen, 1999) and conflict processing (Kanske & Kotz, 2011; Ullsperger, Harsay, Wessel, & Ridderinkhof, 2010). Further, both insula and ACC show activation when the task requires a minimum degree of processing depth (as required by the LDT) (Phan, Wager, Taylor, & Liberzon, 2002). We also more generally predicted better performance and enhanced activation of emotion-related brain regions in response to emotionally-laden words compared to neutral words. Further, we predicted faster LD latencies, higher accuracy and enhanced activation of the classical lexico-semantic neural network in response to words compared to non-words (cf. Fiebach, Friederici, Mueller, & von Cramon, 2002; Price, 2012).

## 2 Method

### 2.1 Participants

Nineteen native British English-speakers from the University of Sussex (10 women, 9 men), aged between 18 and 37 years (mean±SD*=*23.7±5.6 years) took part in the experiment. They were all right-handed with normal or corrected-to-normal vision, had no learning disabilities and took no medication for mood disorders. Participants either received course credits or were paid £10 for their participation. They all gave written informed consent before participating. Three participants were excluded from the fMRI analyses during image processing due to head movement artefacts exceeding 3 mm. Due to failure to record behavioural data from other two participants, only seventeen participants were included in RT and accuracy analyses.

### 2.2 Materials

#### 2.2.1 Word selection and manipulation

One-hundred and seventy-five words were selected from a corpus of English words (Citron et al., 2012), containing subjective ratings for affective features – emotional valence, arousal – and linguistic or more specifically lexico-semantic characteristics – word familiarity, age of acquisition (AoA) and imageability. Seven-point Likert scales were used to quantify the different variables and the extremes were labelled as follows: valence ranged from −3 (very negative) to +3 (very positive); arousal, familiarity and imageability were scaled from 1 (not at all) to 7 (very high); for AoA, age ranges in years were given: 0–2, 2–4, 4–6, 6–9, 9–12, 12–16, older than 16, subsequently recoded in 1-to-7 points. The absolute values of emotional valence were used to form an additional variable called “emotionality”. This variable gives a measure of valence that is independent of the direction of the rating (positive versus negative) and thus provides an absolute measure of the rated emotionality of a word. Length in letters, phonemes, syllables and frequency (spoken and written) were taken from the web-based CELEX (Max Planck Institute for Psycholinguistics, 2001). Written word neighbourhood size (*N*-size) and frequency (*N*-frequency) values were taken from the ELP database (Balota et al., 2007). *N*-size reflects the number of words generated by changing one letter of the target word and *N*-frequency reflects the number of words that share letters with the target word.

The two subjective ratings of interest to testing our hypotheses are emotional valence and arousal. These ratings were used to select 35 positive high-arousal (PH), 35 positive low-arousal (PL), 35 negative high-arousal (NH) and 35 negative low-arousal words (NL). In addition, 35 neutral words were selected, whose arousal level was comparable to the level of low-arousal valenced words.

Descriptive statistics for the 5 conditions are presented in Table 1. Words in all 5 conditions were matched for rated imageability, length in letters, phonemes and syllables, logarithm of frequency of use, word *N*-size and also *N*-frequency (*Fs*(4,170)<2.23, *ns*). Positive and negative high-arousal words were matched for emotionality and for arousal ratings; similarly, positive and negative low-arousal words were also matched (all *ts*(68)<2.02, *ns*). There was no linear correlation between ratings of emotional valence and arousal (*r*=−0.10, *ns*), but a strong quadratic correlation (*r*^2^=0.60, *p*<0.0001), i.e., a correlation between emotionality and arousal. Thus, valence and arousal were manipulated in an orthogonal design.

#### 2.2.2 Pseudoword selection

One-hundred and seventy-five pseudowords were selected from the ARC non-word database (Rastle, Harrington, & Coltheart, 2002). Pseudowords are non-existent words in English that nevertheless follow the orthographic and phonological rules of English. Length of pseudowords ranged between 3–10 letters and 2–8 phonemes. Pseudowords were matched with the 175 words for length in letters *t*(316.11)=0.28, *ns* and number of phonemes *t*(302.21)=1.32, *ns*.

### 2.3 Procedure

The experiment was conducted at the Clinical Imaging Sciences Centre (CISC) at the University of Sussex. The experiment was programmed in Matlab using the Cogent toolbox (Wellcome Laboratory of Neurobiology, http://www.vislab.ucl.ac.uk/cogent.php). Stimulus order and timings were optimised to maximise the statistical efficiency of the task design by using OPTSEQ2 (Dale, 1999) which created a randomised sequence of experimental conditions and null events of varying durations (i.e., jittered). Using this sequence template, 4 different string (word or pseudoword) orders were implemented. The 385 experimental trials lasted 3300–5000 ms, and additional 166 null events lasted 3315–24061 ms.

Participants gave informed consent for the fMRI procedure, following written and oral instructions on how to perform the task. A structural image scan lasting approximately 5 min was acquired before the main experiment. At the beginning of the experiment, 3 filler letter strings were presented, that were later excluded from the analysis. The experiment was divided into 3 sessions containing 196, 196 and 197 events each (fillers, strings, null events). In between sessions, the scanner was stopped and participants had a few minutes to rest.

Each trial began with a central fixation cross, visible for 1300–2999 ms (jittered interval length). Subsequently, a string appeared for 250 ms, followed by a 100-ms blank screen, then by a question mark, which prompted a response and remained present until a response was given. Participants were required to read the letter strings and decide whether the stimulus was an English word or not, as accurately and as quickly as possible. A response pad with two buttons corresponding to “yes/no” answers was provided and the button configuration was counterbalanced across participants. A fixed time interval of 1650 ms between the onset of the question mark and presentation of the next trial was used to ensure that the trial duration was at least 3300 ms (corresponding to the TR). The mean trial length was 3802 ms (SD=288, range=3300–4299 ms). Overall, the experiment lasted approximately 1 h and 40 min, including preparation, structural scanning, 55 min of functional scanning time and debriefing. Approximately 1000 functional volumes per participant were acquired.

### 2.4 MRI data acquisition and preprocessing

Hemodynamic responses were acquired by means of a 1.5 T scanner (Siemens Avanto) with a standard head matrix coil. For each participant, full-brain, T1-weighted structural scans were acquired: 192 slices, 0.9 mm thick with a 15° flip angle, 0.9 mm isotropic voxels without gap, MPRAGE, TR 11.6 s, TE 4.4 s, 300 ms inversion time, 250×250 matrix per slice. For functional images, 36 slices were acquired, 3 mm thick with 90° flip angle, 3×3×3.75 mm voxels with gap, TR 3300 ms, TE 50 ms, 64×64 mm matrix per slice.

Image processing and statistical analyses were performed using SPM5 (Wellcome Trust Centre, http://www.fil.ion.ucl.ac.uk/spm/), employing spatial realignment and sequential coregistration (6-parameter rigid body spatial transformation). Structural images were segmented into grey and white matter and cerebrospinal fluid (CSF) and iteratively normalised to standard space (Montreal Neurologic Institute, MNI). Transformation parameters for structural images were then applied to functional images. Subsequently, functional volumes were spatially smoothed with an 8-mm Gaussian kernel to adjust for between-participants anatomical differences. The first 5 functional volumes were discarded to allow for equilibration of net magnetisation. In order to detect further movement artefacts after realignment, the software ArtRepair (http://cibsr.stanford.edu/publications/publications.htm) was used (*z* threshold=11, movement threshold=3) and additional movement regressors for outliers were created.

### 2.5 Statistical analysis

#### 2.5.1 Behavioural data

Lexical decision latencies and accuracy were analysed by means of 3 different designs: lexicality (words, pseudowords), emotionality (neutral, positive, negative) and valence (positive, negative) by arousal (high, low). As a standard in psycholinguistic research (cf. Clark, 1973), we conducted analyses by participant (1 subscripted), in which the raw data are averaged within each experimental condition and compared in a within-subjects design, as well as analyses by item (2 subscripted), in which the data points for each single stimulus are averaged across participants and the words belonging to each condition are compared in a between-subjects design. More specifically, we used *t*-tests or ANOVAs depending on the number of levels for each factor. For the lexicality design, we used one-directional *t*-tests. If the main emotionality effect and the valence by arousal interaction were significant, one-directional planned contrasts between neutral vs. emotionally-valenced words and between words eliciting conflicting vs. congruent tendencies were performed. In case of violation of the sphericity assumption, we used the Greenhouse-Geiser correction and in case of inhomogeneity of variances, we used Welch statistics. Only correctly-responded trials were included in RT analyses and, for each participant, outlier correction of RTs±3 SDs was applied. A significance level of *P*<0.05 was used.

#### 2.5.2 Neuroimaging data

A general linear model was used in an event-related design. Hemodynamic responses were time-locked to the stimulus onset and convolved with the canonical hemodynamic response function of SPM5. Six separate regressors were used to model each condition: pseudowords, PH, PL, NH, NL and neutral words. In order to account for signal changes not related to the conditions of interest, six head movement regressors were added as covariates. For some participants, additional artefact regressors, created with the ArtRepair toolbox, were added to the model.

As with the behavioural analyses, lexicality, emotionality and valence by arousal factorial designs were employed for the imaging data, by defining *T*-contrasts for each participant. For the lexicality design, words were contrasted with pseudowords. For the emotionality design, valenced words were contrasted with neutral words; in addition, positive and negative words were separately contrasted with neutral words. For the valence by arousal design, main effects were tested by contrasting positive and negative words, as well as high- and low-arousal words. The interaction between factors was tested by contrasting PH and NL words with PL and NH words. Further pair-wise comparisons were also performed. At the second (group) level analysis, one-sample *t*-tests in both directions were performed using the contrast images created at the first (single-participant) level. For significance levels, a voxel-level threshold of *P*<0.001 uncorrected was chosen, along with a cluster-level threshold of *P*<0.05, corrected for family-wise error (FWE).

## 3 Results

### 3.1 Behavioural results

**Fig. 1:**
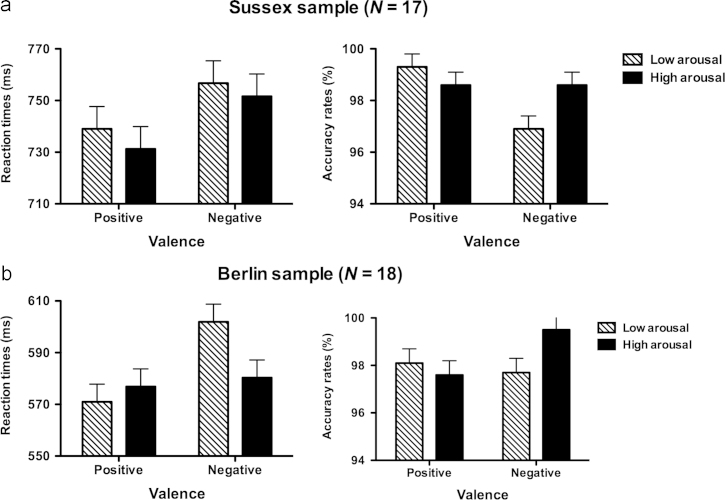
Descriptive statistics of mean reaction times and mean accuracy rates for the valence by arousal design, analyses by item: (a) Sussex sample and (b) Berlin sample. Error bars represent standard errors of the mean.

Mean accuracy overall was 97%. Descriptive statistics are reported in Table 2 and displayed in Fig. 1a.

#### 3.1.1 Lexicality

Words were responded to significantly faster (*t*_1_(16)=3.33, *p*<0.01; *t*_2_(335.36)=15.36, *p*<0.0001) and more accurately (*t*_1_(16)=3.58, *p*<0.01; *t*_2_(373.60)=2.49, *p*<0.01) than pseudowords (see Table 2).

#### 3.1.2 Emotionality

RT results revealed a main effect of emotionality (*F*_1_(2,32)=5.22, *p*<0.05), not confirmed by the item analysis (*F*_2_(2,172)=2.22, *p*=0.11). A trend toward slower RTs for neutral than emotionally-valenced words was found (*F*_1_(1,16)=3.01, *p*=0.051); further comparisons revealed significantly slower RTs for negative than neutral words (*F*_1_(1,16)=8.66, *p*<0.01). See Table 2 for descriptive statistics.

Accuracy results also revealed a significant effect of emotionality, in both participant and item analyses (*F*_1_(2,32)=9.23, *p*<0.001; *F*_2_(2,172)=4.10, *p*<0.05). Planned contrasts revealed significantly lower accuracy for neutral than emotionally-valenced words (*F*_1_(1,16)=11.08, *p*<0.01; *t*_2_(172)=2.42, *p*<0.01).

#### 3.1.3 Valence by arousal

RT results revealed a main effect of valence with faster responses to positive than negative words (*F*_1_(1,16)=8.18, *p*<0.05; *F*_2_(1,136)=4.85, *p*<0.05). No main effect of arousal (*F*_1_(1,16)=1.85, *ns*; *F*_2_(1,136)=0.55, *ns)* and no interaction (*F*_1_(1,16)=0.65, *ns*; *F*_2_(1,136)=0.02, *ns*) were found (see Fig. 1a).

Accuracy results revealed a main effect of valence with higher accuracy for positive than negative words (*F*_1_(1,16)=5.34, *p*<0.05; *F*_2_(1,136)=4.93, *p*<0.05), accompanied by a significant interaction (*F*_1_(1,16)=8.13, *p*<0.01; *F*_2_(1,136)=4.93, *p*<0.05), whereby words eliciting conflicting approach-withdrawal orientations (PH and NL conditions) showed lower accuracy than words eliciting congruent orientations (PL and NH) (*t*_1_(16)=2.85, *p*<0.01; *t*_2_(118.38)=2.19, *p*<0.05) (see Fig. 1a).

### 3.2 Functional imaging results

#### 3.2.1 Lexicality

Several brain regions were significantly activated for the contrast words>pseudowords (refer to Table 3 for a detailed list). Increased activations for words were found in the left inferior and superior frontal gyri (IFG, SFG) and in the left dorsomedial prefrontal cortex (dmPFC). Clusters of activation were also found bilaterally within the middle and superior temporal gyri (MTG, STG), and in the right middle cingulate cortex (CC). These areas are known to be part of a general language network (cf. Ferstl, Neumann, Bogler, & von Cramon, 2008), but more specifically, they are activated in response to the retrieval of lexical and semantic word representations (Fiebach et al., 2002; Price, Wise, & Frackowiak, 1996).

#### 3.2.2 Emotionality

**Fig. 2:**
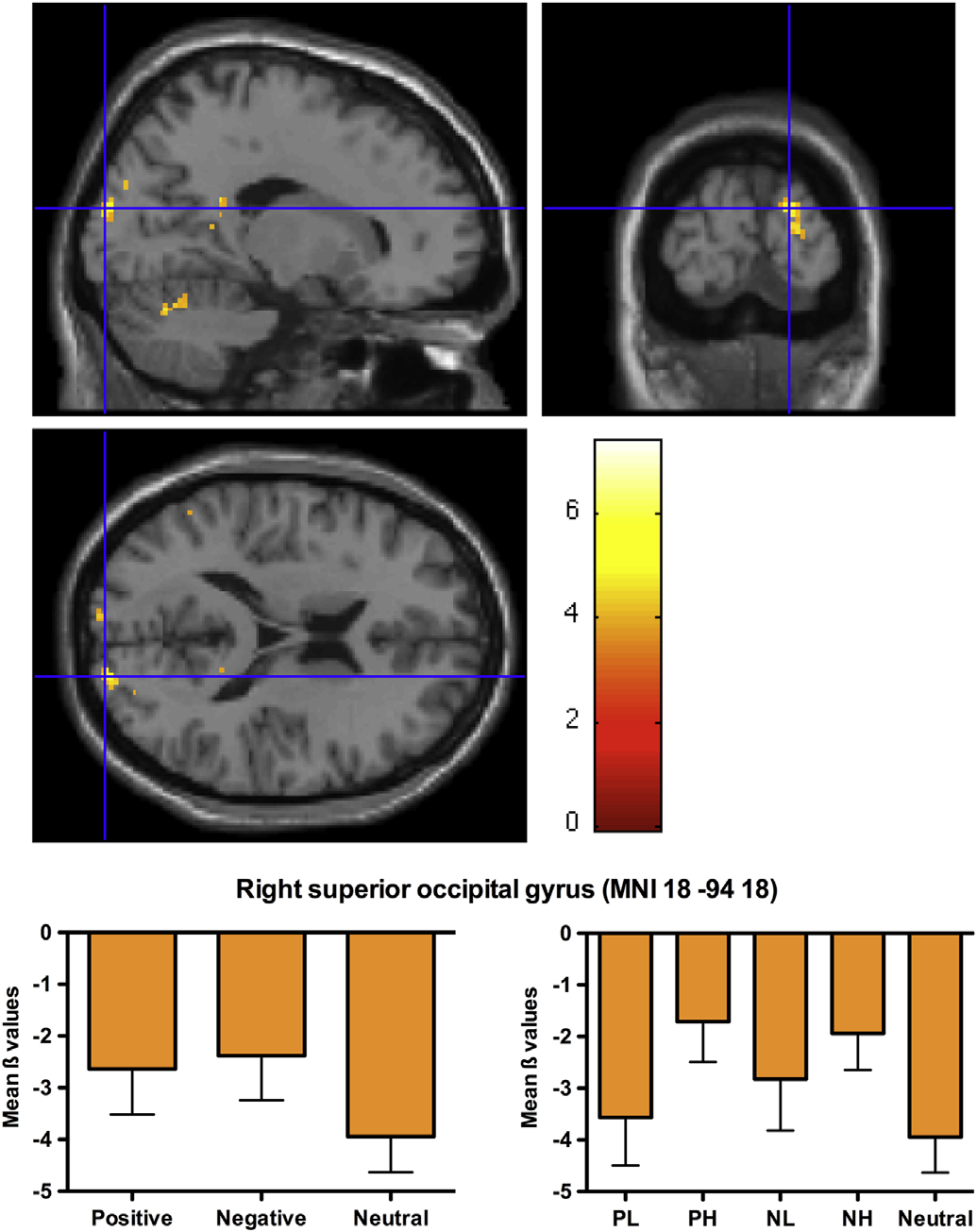
Regions showing significant BOLD signal changes to emotional words compared to neutral words. Less decrease in activation for emotional words was found significant in the left extra-striate cortex. A small cluster of activation is also visible in the right homologous region, even though not significant. The left diagram shows the signal change (ß values) for positive, negative and neutral words; these conditions are broken down by valence and arousal in the right diagram.

The contrast between emotionally-valenced and neutral words (positive+negative>neutral words) revealed a cluster of significant activation in the right superior occipital gyrus (SOG) and cuneus, both part of the extra-striate cortex (see Table 4 and Fig. 2). A similar cluster was also significant for negative words compared to neutral ones, whereas activation of the SOG did not reach corrected cluster level significance in the contrast positive vs. neutral words.

#### 3.2.3 Valence by arousal

**Fig. 3:**
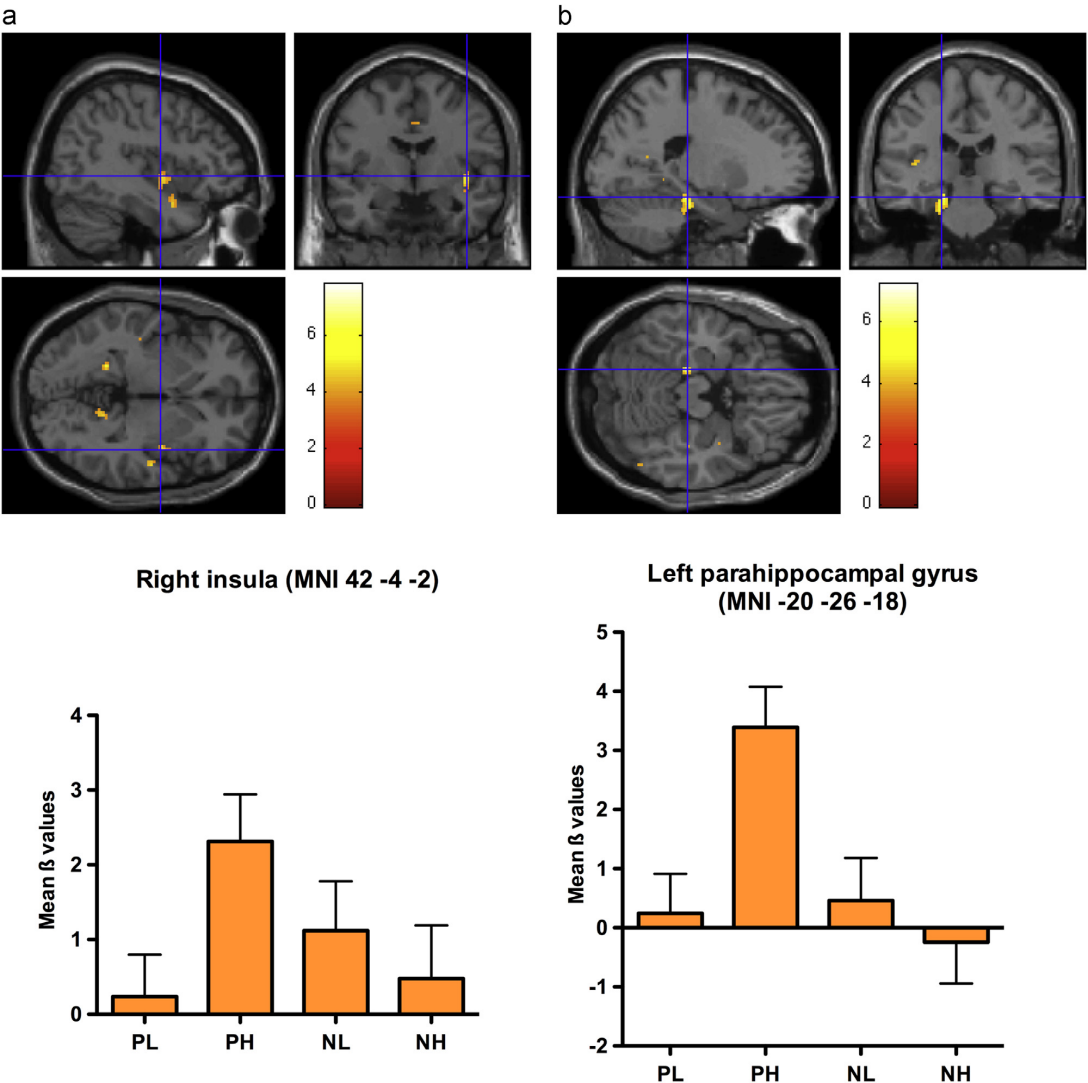
(a) Regions showing significant BOLD signal changes to positive high-arousal (PH) and negative low-arousal (NL) words compared to positive low-arousal (PL) and negative high-arousal (NH) words. Increase in activation for the former two conditions was found in the right insula, also significantly activated in the pair-wise comparison PH>PL. (b) In addition, the latter contrast showed significant activation of the left parahippocampal gyrus. Please refer to Table 4 for exact MNI coordinates. Diagrams of increase or decrease in activation (ß values) are reported for the 4 conditions. Error bars represent standard errors of the mean.

A significant cluster of activation in the right insula extending to the superior temporal gyrus (STG) was observed in response to the interaction between valence and arousal (PH+NL>PL+NH). As can be seen in Fig. 3a, this region showed increased activation for PH and NL conditions, stronger for the former, and very little response to PL and NH conditions. A second cluster within the left posterior insula did not reach corrected cluster level significance (*T*=6.12, *p*=0.078). No main effects of either valence or arousal were found.

Further planned pair-wise comparisons extended the results found for the interaction by showing a bigger cluster of activation in the right insula and STG for the contrast PH>PL (see Table 4). Again, a cluster of activation in the left posterior insula was observed at a level just below corrected cluster level significance. In addition, a significant activation of the left parahippocampal gyrus was found, with increased activation for PH, but no response to PL (see Fig. 3b). No other pair-wise comparisons showed significant clusters of activation. Nevertheless, clusters in the left parahippocampal gyrus and in right STG were visible for the contrast PH>NH and a cluster in the right pulvinar of the thalamus was apparent for the contrast NL>NH (see Table 4).

### 3.3 Post-hoc collection of behavioural data from an independent sample

The most important result of our study, namely the interaction between valence and arousal dimensions in insular cortex, was supported by a similar interactive pattern in the accuracy rates, but not in the reaction times. In our view, behavioural data collected in the scanner might be more noisy and have higher variance than data collected while sitting in front of a computer screen, because of greater fatigue (e.g., scanner noise, movement constraints, etc.). Therefore, we decided to conduct a second, independent behavioural replication study.^1^

#### 3.3.1 Participant sample

Eighteen native English-speakers living in the Berlin area (8 women, 10 men), aged between 18 and 30 years (mean±SD*=*23.9±3.2 years) took part in the experiment. Participants came from different English-speaking countries. They all had normal or corrected-to-normal vision and 16 of them were right-handed. Participants were paid 5€ for their participation. They all gave written informed consent before participating.

#### 3.3.2 Methods

The experiment was conducted in a quiet room, where participants sat in front of a computer screen. They responded by pressing two buttons highlighted on the keyboard. All other details regarding the programming of the experiment, the timing of stimulus presentation, the randomisations, as well as the data analyses, are identical to the ones in the original experiment.

#### 3.3.3 Group comparison

The Berlin sample showed high mean accuracy overall (97%), not different from the accuracy of the Sussex sample (*t*_1_(33)=0.02, *ns*), but significantly faster RTs (*t*(25.83)=5.20, *p*<0.0001) and much lower variance. Please refer to Table 2 for descriptive statistics.

#### 3.3.4 Berlin sample: lexicality

As in the previous sample, words were responded to significantly faster (*t*_1_(17)=7.51, *p*<0.0001; *t*_2_(348)=12.38, *p*<0.0001) and more accurately (*t*_1_(17)=1.66, *p*=0.06; *t*_2_(321.60)=2.64, *p*<0.01) than pseudowords (see Table 2). The difference in accuracy was smaller than in the Sussex sample, possibly due to the fact that the Berlin sample came from a more heterogeneous language background. In fact, some participants expressed their awareness of the British spelling and the possibility of not knowing some specific British words.

#### 3.3.5 Berlin sample: emotionality

As in the Sussex sample, RT results revealed a main effect of emotionality, this time consistent across participant and item analyses (*F*_1_(1.32,22.37)=4.31, *p*<0.05; *F*_2_(2,172)=3.63, *p*<0.0.05). Planned contrasts revealed no difference between neutral and emotionally-valenced words (*F*_1_(1,17)=0.46, *ns*; *t*_2_(172)=1.36, *p*=0.09), but further comparisons showed significantly slower RTs for neutral than positive words (*F*_1_(1,17)=4.39, *p*=0.05; *t*_2_(172)=2.19, *p*<0.05). See Table 2 for descriptive statistics.

Accuracy results also revealed a main effect of emotionality (*F*_1_(2,34)=8.92, *p*<0.001; *F*_2_(2,172)=3.60, *p*<0.05), with lower accuracy for neutral than emotionally-valenced words (*F*_1_(1,17)=13.31, *p*<0.001; *t*_2_(172)=2.56, *p*<0.01), confirming the results from the Sussex sample.

#### 3.3.6 Berlin sample: valence by arousal

RT results confirmed a main effect of valence, with faster responses to positive than negative words (*F*_1_(1,17)=22.46, *p*<0.0001; *F*_2_(1,136)=6.29, *p*<0.05). Most importantly, a significant interaction between valence and arousal was found (*F*_1_(1,17)=9.64, *p*<0.01; *F*_2_(1,136)=4.02, *p*<0.05), whereby words eliciting conflicting approach-withdrawal tendencies were responded to more slowly than words eliciting congruent approach or withdrawal tendencies (*t*_1_(17)=3.11, *p*<0.01; *t*_2_(127.16)=1.97, *p*<0.05). A trend toward a main effect of arousal was observed only in the participant analysis (*F*_1_(1,17)=3.31, *p*=0.09; *F*_2_(1,136)=1.31, *ns*). Please refer to Fig. 1b for descriptive statistics.

Accuracy results confirmed a significant interaction between valence and arousal (*F*_1_(1,17)=3.17, *p*<0.05; *F*_2_(1,136)=4.42, *p*<0.05), whereby words eliciting conflicting orientations showed lower accuracy than words eliciting congruent orientations (*t*_1_(17)=1.78, *p*<0.05; *t*_2_(137.97)=2.10, *p*<0.05). As in the previous sample, no main effects of valence (*F*_1_(1,17)=0.49, *ns*; *F*_2_(1,136)=1.52, *ns*) or arousal (*F*_1_(1,17)=0.66, *ns*; *F*_2_(1,136)=1.39, *ns*) were found.

## 4 Discussion

The present study examined how emotional valence and arousal affect hemodynamic brain responses during implicit emotion word processing, within a framework that predicts interactive effects (Robinson et al., 2004). To this end, we employed a lexical decision task and manipulated valence and arousal dimensions orthogonally, by controlling for other lexico-semantic variables that are known to affect written word recognition.

Our main finding, in line with our first hypothesis, was an interaction between the two dimensions of emotion, expressed via increased neural responses within right insular cortex to stimuli eliciting incongruent approach-withdrawal tendencies (PH and NL words, e.g., *rollercoaster* and *weak*, respectively) compared to stimuli eliciting congruent approach vs. withdrawal tendencies (PL and NH words, e.g., *flower* and *bomb*, respectively). In addition, pairwise comparisons showed increased activation in the very same region as well as in the left parahippocampal gyrus for the contrast PH>PL. The interaction at the neural level was supported by behavioural data showing slower LD latencies and lower accuracy for stimuli eliciting conflicting orientations, in line with previous behavioural results (Citron et al., under review; Robinson et al., 2004).

Insular cortex, and more specifically anterior insula, is responsible for the integration of afferent information about the physiological state of the body with on-going cognitive and evaluative processes (Critchley et al., 2004; Gray, Harrison, Wiens, & Critchley, 2007). Initial somatic afferent representations within the posterior insula may underlie consciously accessible feeling states following integrative processing within anterior insula regions: this integration gives rise to emotional awareness (cf. Craig, 2009; Critchley et al., 2004; Damasio et al., 2000). Lateralisation of insular functions has also been proposed (Craig, 2005), whereby right anterior insula is activated by homoeostatic afferents associated with sympathetic functions (e.g., pain) or “energy expenditure”, whereas left anterior insula activation is associated with the parasympathetic system or “energy enrichment” (e.g., nourishing during quiescence).

In our study, implicit integration of conflicting approach-withdrawal tendencies elicited by specific emotionally-laden stimuli was processed in the right insula, consistent with increased sympathetic arousal, and suggesting that these stimuli demand more energy in order to be processed. Further, the proposed functional role of insula suggests that both automatic reaction tendencies and cognitive stimulus evaluation were involved.

Our finding lends support to the multidimensional model proposed by Robinson et al. (2004) and confirms previous findings of interactive effects of valence and arousal during implicit emotion processing (Citron et al., under review; Eder & Rothermund, 2010; Feng et al., 2012; Hofmann et al., 2009; Larsen et al., 2008). Moreover, this result extends previous behavioural and ERP findings by suggesting a possible functional neural correlate of the integration of pre-attentive approach-withdrawal tendencies elicited by salient stimuli, namely the right insula.

The model proposed by Robinson et al. (2004) differs from previous models in that it predicts approach vs. withdrawal tendencies for low vs. high arousal independently of whether the stimulus is positive or negative. For example, other models of emotion processing predict appetitive behaviour toward highly arousing positive stimuli such as sugary food or sexually attractive pictures, given that they are positive in valence (e.g., Lang et al., 1997; Lang, Bradley, & Cuthbert, 1999). The prediction of conflicting approach-withdrawal tendencies for PH stimuli made by Robinson et al. is based on distinct effects of valence and arousal dimensions and cannot be predicted by previous models, which associate increase in (positive or negative) valence with a necessary increase in emotional arousal (Bradley & Lang, 1999; Lang et al., 1997). In this respect, empirical support for partial distinction (e.g., Ito, Larsen, Smith, & Cacioppo, 1998) and a non-perfect correlation between these two variables (Citron et al., 2012) allows room for interactive effects, that can only be predicted by an interactive model. As an example, *rollercoaster* represents something positive and exciting one might want to approach, but also something very intense, that might elicit withdrawal.

The interaction between emotional variables elicited no activation in the ACC. This null finding is consistent with the notion that the ACC is typically engaged by emotional (or cognitive) conflict elicited by the task requirements (cf. Kanske & Kotz, 2011; Ullsperger et al., 2010); for example, in the go/no-go or in the Stroop task, the participant needs to put effort in order to avoid a strong, automatic response tendency and to successfully perform the task. In such situations, the individual is aware of the conflict and explicitly acts in order to solve it (Ullsperger et al., 2010). This importantly differs from the type of conflict we propose in our study, which is induced by the implicit integration of conflicting stimulus-driven response tendencies to emotional valence and arousal.

Besides the insula, the anterior portion of the right superior temporal gyrus (STG), also referred to as anterior temporal lobe (ATL), also showed enhanced activation in the interaction as well as in the PH>PL contrast. Bilateral ATL activation is associated with semantic/conceptual categorisation (Rogers et al., 2006) as well as comprehension of coherent, comprehensible text (Ferstl et al., 2008). More specifically, right ATL showed enhanced activation during comprehension of emotionally and chronologically inconsistent stories compared to consistent ones (Ferstl, Rinck, & von Cramon, 2005), thus suggesting a possible role of this region in making sense of emotionally incongruent information. Besides language, ATL is also involved more generally in social-emotional cognition (cf. Wong & Gallate, 2012); for example, the right anterior STG contributes to encoding facial expressions, as it responds to dynamic changes in face features (Haxby, Hoffman, & Gobbini, 2002) and is more strongly activated in response to judgment of emotion from facial expression than to simple face detection (Streit et al., 1999). Thus this region is also involved in decoding the emotional content of visual information.

Activation of the parahippocampal gyrus in response to PH compared to PL words was also not specifically predicted but is not surprising; in fact, this region is part of the Papez circuit, one of the major pathways of the limbic system, involved in the cortical control of emotion, as well as in maintaining novel information in working memory (cf. Bear, Connors, & Paradiso, 2006; Hasselmo & Stern, 2006).

PH words elicited the highest increases in activation in the right insula, both in the contrast and in the interaction. These words, even though matched for emotionality and arousal level with the NH words, might represent the most strongly emotionally-laden words in our study. In fact, negative stimuli are naturally more intense than positive ones (cf. Bradley & Lang, 1999; Citron et al., 2012). In our manipulation, highly arousing negative words such as *rape*, *war* or *death* had to be excluded in order to obtain a good matching with the corresponding positive words.

A second important finding, in line with our general second hypothesis, was significant activation within the left extra-striate cortex in response to emotionally-valenced words compared to neutral words, in line with previous research (Compton et al., 2003; Herbert et al., 2009). This result is interesting for two reasons: First of all, it suggests enhanced processing (or stronger attention capture) of emotionally salient stimuli (Compton et al., 2003; Kissler et al., 2007) in regions that are functionally associated with perceptual, i.e., visual processing; Further, source localisation techniques found the left extra-striate cortex to be the source of an early ERP component (200–300 ms, with posterior scalp distribution) associated with implicit emotion word processing, namely the early posterior negativity (EPN; Kissler et al., 2007). Thus, the emotional connotation of verbal stimuli seems to modulate not only emotion-related processes, but also early, perception-related processes, during reading.

The fact that no clusters of activation for the contrast emotional>neutral in emotion-related cortical areas (e.g., OFC, amygdala) were found might be due to the relatively small difference in arousal level between the two conditions. Our neutral words had the same arousal level as half of our valenced words and, as can be seen in the bottom right diagram of Fig. 2, the effect was mainly driven by high-arousal valenced words (i.e., PH, NH), which showed the least decrease of activation in this region. Previous studies reporting OFC activation compared highly arousing positive and negative words with neutral ones (e.g., Kuchinke et al., 2005). Further, OFC activation is typically elicited by tasks that require deep encoding of the emotional material, such as valence decision (Dolcos, LaBar, & Cabeza, 2004; Small et al., 2003), associated with retrieval of emotional memories (Posner et al., 2009), or self-referential tasks (Lewis et al., 2007).

Activation of the amygdala has also been typically reported in studies employing highly arousing positive and negative words (Hamann & Mao, 2002; Lewis et al., 2007) and low-arousal neutral words (Hamann & Mao, 2002). Further, this region is associated with perceptual processing of emotionally-laden material (Garavan, Pendergrass, Ross, Stein, & Risinger, 2001) and its activation may be attenuated or suppressed by cognitively demanding tasks (Phan et al., 2002). In fact, amygdala activation was absent in Kuchinke et al. (2005), who employed a LDT, but was instead reported by Herbert et al. (2009) during silent reading. In our study, the subtle manipulation of arousal levels within valenced words as well as between valenced and neutral words, along with the employment of a LDT, might have hindered amygdala activation.

As an additional note, we would like to mention the fact that single words elicit little BOLD-signal response compared to emotional pictures (Citron, 2012) and only a few neuroimaging studies on implicit emotion word processing exist in the literature (i.e., Herbert et al., 2009; Kuchinke et al., 2005; Lewis et al., 2007). See also Schlochtermeier et al. (2013) for a detailed investigation of the effects of the type of emotional material (verbal vs. pictorial) and its visual complexity.

At the behavioural level, we report better performance for valenced than neutral words, in line with previous literature (e.g., Kousta et al., 2009). Furthermore, faster RTs and higher accuracy for positive than negative words are in line with Kuchinke et al. (2005), who interpreted this effect in light of a more interconnected network of lexical and semantic representations for positive words, therefore making their processing easier (cf. Ashby, Isen, & Turken, 1999). Finally, the lack of an arousal effect within positive and negative words can again be attributed to the relatively small difference in arousal level between high- and low-arousal valenced words. Furthermore, arousal effects *within* valenced words are not well-established at the behavioural level (cf. Bayer et al., 2012; Hofmann et al., 2009).

The behavioural data collected outside of the scanner showed stronger and more consistent effects than the data collected while scanning, i.e., most effects were significant in both participant and item analyses and the interactive pattern between valence and arousal was replicated in the reaction times, beyond accuracy rates. These results are not surprising, given the fact that performance in the scanner might be affected by a number of factors (e.g., noise, strain, movement constraints) and therefore lead to increased noise and variance in the data. In fact, the Sussex sample showed significantly slower RTs and much larger variance than the Berlin sample. Accuracy in a LDT is typically very high and in this study it was not affected by the environmental conditions.

A possible limitation of our 2×2 design is the fact that the interactive pattern of effects may be driven by other variables. For example, the fact that the difference in BOLD response between PH and PL conditions is larger than the difference between the corresponding negative conditions, along with the significant pair-wise comparison, may suggest that the interactive effect found is actually driven by an arousal effect within positive words. We cannot currently rule out this interpretation, but only suggest that future confirmation of this pattern within positive and negative stimuli is needed. One promising approach would be to use a more naturalistic word selection. In particular, selecting highly arousing negative words – that do not need to match positive words in arousal level – might induce a larger difference between high- and low-arousal valenced words. In fact, first results of a pilot study using German words yields a clear interactive pattern, weighting on both positive and negative words.

Finally, our experimental design could also be used to conduct research on mood disorders, such as anxiety or depression, that typically disrupt processing of emotionally salient stimuli (cf. Mathews & MacLeod, 1994)

### 4.1 Conclusions

The present study provides new empirical evidence from fMRI in support of a multidimensional, interactive model of emotion processing (Robinson et al., 2004), whereby valence and arousal dimensions affect the processing of emotional stimuli interactively, i.e., positive high-arousal and negative low-arousal words elicit conflicting approach-withdrawal tendencies and therefore require more processing resources than positive low-arousal and negative highly arousing words, that elicit congruent approach vs. withdrawal tendencies, respectively.

Our findings add to previous research showing that interactive effects arise during implicit processing of a stimulus′ emotional content (e.g., Eder & Rothermund, 2010; Feng et al., 2012) and propose for the first time a specific neural correlate reflecting the integration of pre-attentive approach-withdrawal tendencies elicited by salient stimuli, namely the right insular cortex.

Finally, our findings support the claim that emotional variables affect even highly abstract cognitive processes such as reading, beyond other well-known lexico-semantic variables, and this could have implications for formal education in school, as well as the assessment and diagnosis of mood disorders.

## Figures and Tables

**Table 1:** Descriptive statistics for selected words. Means, minimum and maximum scores for each condition are reported. Emotionality refers to the absolute valence ratings. Freq_log refers to the logarithm of word frequency, N-size and N-frequency to neighbourhood size and frequency, respectively.

	**Positive, high arousal**			**Negative, high arousal**			**Positive, low arousal**			**Negative, low arousal**			**Neutral, low arousal**		
	**Mean**	**Min**	**Max**	**Mean**	**Min**	**Max**	**Mean**	**Min**	**Max**	**Mean**	**Min**	**Max**	**Mean**	**Min**	**Max**
**Emotionality**	1.92	1.01	2.52	1.77	1.17	2.61	1.46	1.04	1.90	1.33	0.89	2.02	0.57	0.04	0.85
**Em. Valence**	1.92	1.01	2.52	−1.77	−2.61	−1.17	1.46	1.04	1.90	−1.33	−2.02	−0.89	0.19	−0.85	0.85
**Arousal**	4.45	4.00	5.35	4.60	4.06	5.41	3.41	2.59	3.88	3.52	2.24	4.42	3.32	2.79	4.15
**Imageability**	4.31	2.51	6.37	3.88	2.20	6.51	3.73	2.07	6.71	3.44	1.96	6.48	3.84	2.05	6.44
**Letters**	7	4	12	7	3	11	7	3	11	7	3	13	6	3	12
**Phonemes**	6	3	13	5	2	10	5	2	10	6	3	12	5	2	12
**Syllables**	2	1	5	2	1	4	2	1	5	2	1	4	2	1	4
**Frequency**	42	0	172	33	2	148	49	1	272	44	1	267	85	1	996
**Freq_log**	3.01	0.00	5.15	2.78	0.69	5.00	3.15	0.00	5.61	2.91	0.00	5.59	3.59	0.00	6.90
**N-size**	2	0	12	3	0	18	3	0	21	5	0	23	4	0	34
**N-freq**	6	3	9	6	0	9	6	1	8	6	0	9	6	1	9

**Table 2:** Descriptive statistics of the behavioural results for lexicality and emotionality designs, analyses by item. RT=reaction time; SE=standard error of the mean.

**Design**	**Condition**	**Sussex sample, inside the scanner**		**Berlin sample, outside the scanner**	
		Mean RT in ms (SE)	Mean accuracy % (SE)	Mean RT in ms (SE)	Mean accuracy % (SE)
Lexicality	Words	**744.07** (3.72)	**98** (0.3)	**586.29** (3.07)	**98** (0.4)
	Pseudowords	**833.02** (4.44)	**96** (0.5)	**643.48** (3.45)	**96** (0.5)

Emotionality	Neutral	**740.02** (11.46)	**96** (1.4)	**593.79** (9.03)	**96** (1.5)
	Positive	**735.16** (6.14)	**99** (0.3)	**573.90** (4.79)	**98** (0.4)
	Negative	**754.18** (6.01)	**98** (0.4)	**591.11** (5.02)	**99** (0.4)

**Table 3:** Regions showing significant BOLD signal change to words compared to pseudowords.

**Words**>**Pseudowords**							
**Lobe**	**Hemi.**	**Region**	**Cluster size**	***T***	***x***	***y***	***z***
Frontal	L	Dorso-medial prefrontal cortex	279*	5.87	−**6**	**56**	**32**
		Superior medial frontal gyrus		4.82	−**8**	**60**	**22**
		Superior frontal gyrus		4.24	−**14**	**46**	**34**

	L	Inferior frontal gyrus	84*	4.98	−**48**	**24**	**10**
		Inferior frontal gyrus		4.49	−**56**	**22**	**22**

Temporal	L	Middle temporal gyrus	121*	5.85	−**54**	−**14**	−**16**
		Superior temporal pole		5.50	−**54**	**6**	−**14**
	R	Superior temporal gyrus	93*	7.17	**54**	−**10**	−**12**
	L	Middle temporal gyrus	664*	6.00	−**54**	−**50**	**8**
		Middle temporal gyrus		5.27	−**40**	−**50**	**16**
		Middle temporal gyrus		5.23	−**60**	−**60**	**0**
	R	Middle temporal gyrus	332*	6.28	**46**	−**60**	**14**
		Middle temporal gyrus		6.05	**58**	−**48**	**2**

Cingulate	R	Middle cingulate cortex	85*	5.12	**4**	−**16**	**42**
		Supplementary motora area		5.08	**8**	−**22**	**48**

Hemi.=hemisphere, L=left, R=right; cluster size is in voxels, *T*=peak *t*-value; *x*, *y*, *z*=MNI stereotactic space coordinates.

⁎ Significant clusters (with correction).

**Table 4:** Regions showing significant BOLD signal change in the emotionality and valence by arousal designs. Significant clusters with correction are marked with an asterisk. Additional non-significant clusters exceeding an extent threshold of at least 45 contiguous voxels are reported for completeness. When identifiable, Brodmann areas (BA) were reported along with the cortical region.

**Contrasts, followed by Hemi.**	**Region**	**Cluster size**	***T***	***x***	***y***	***z***
**Emotional**>**neutral words**						
R	Superior occipital gyrus (BA 18)	84*	5.56	**18**	−**94**	**18**
	Cuneus		4.91	22	−96	10

**Negative**>**neutral words**						
R	Superior occipital gyrus (BA 19)	86*	5.87	**18**	−**92**	**20**
	Cuneus		5.55	12	−86	26
R	–	52	4.62	30	−40	12
	Precuneus		4.61	22	−46	12
	Cerebellum	50	5.36	20	−62	−22

**Positive**>**neutral words**						
R	Superior occipital gyrus	57	4.84	22	−94	8
	Superior occipital gyrus (BA 19)	4.55	20	−94	20	20

**Interaction valence by arousal: PH+NL**>**PL+NH**						
R	Insula (BA 13)	81*	5.35	**42**	−**4**	−**2**
	Superior temporal gyrus		4.72	44	6	−24
L	Rolandic operculum	68	6.12	−42	−22	20
	Posterior insula		5.10	−42	−16	26
	Posterior insula (BA 13)		4.02	−36	−22	12
R	Cerebellum	46	7.79	32	−48	−32

**PH**>**PL**						
R	Insula	119*	5.89	**42**	−**4**	−**2**
	Superior temporal gyrus		5.04	52	−12	−2
	Insula		4.61	40	−6	−14
L	Parahippocampal gyrus (BA 35)	81*	7.22	−**20**	−**26**	−**18**
L	Posterior insula	47	4.81	−38	−16	0
	Posterior insula (BA 13)		4.67	−32	−22	14
	Superior temporal gyrus		4.38	−40	−26	8

**NL**>**NH**						
R	Pulvinar (thalamus)	50	5.79	16	−32	8
	Pulvinar (thalamus)		4.39	22	−32	2

**PH**>**NH**						
R	Superior temporal gyrus	67	6.26	46	−2	−8
L	Parahippocampal gyrus	46	6.44	−18	−26	−22

Hemi.=hemisphere, L=left, R=right; cluster size is in voxels, *T*=peak *t*-value; *x*, *y*, *z*=MNI stereotactic space coordinates.

⁎ Significant clusters (with correction).

## Footnotes

1 We would like to thank an anonymous reviewer for this suggestion.

## References

[bib1] Ashby F.G., Isen A.M., Turken U. (1999). A neuropsychological theory of positive affect and its influence on cognition. Psychological Review.

[bib2] Balota D.A., Yap M.J., Cortese M.J., Hutchinson K.A., Kessler B., Loftis B., Treiman R. (2007). The English lexicon project. Behavior Research Methods.

[bib3] Bayer M., Sommer W., Schacht A. (2012). P1 and beyond: Functional separation of multiple emotion effects in word recognition. Psychophysiology.

[bib4] Bear M.F., Connors B.W., Paradiso M.A. (2006). Neuroscience: Exploring the brain.

[bib5] Botvinick M., Nystrom L.E., Fissell K., Cater C.S., Cohen J.D. (1999). Conflict monitoring versus selection-for-action in anterior cingulate cortex. Nature.

[bib6] Bradley M.M., Lang P.J. (1999). Affective norms for English words (ANEW): Simuli, instruction manual and affective ratings.

[bib7] Brooks J.C.W., Zambreanu L., Godinez A., Craig A.D.B., Tracey I. (2005). Somatotopic organisation of the human insula to painful heat studied with high resolution functional imaging. NeuroImage.

[bib8] Citron F.M.M. (2012). Neural correlates of written emotion word processing: A review of recent electrophysiological and hemodynamic neuroimaging studies. Brain and Language.

[bib10] Citron F.M.M., Weekes B.S., Ferstl E.C. (2013). Effects of valence and arousal on written word recognition: Time course and ERP correlates. Neuroscience Letters.

[bib9] Citron F.M.M., Weekes B.S., Ferstl E.C. (2014). Arousal and emotional valence affect written word recognition in an interactive way. Manuscript under review.

[bib11] Citron F.M.M., Weekes B.S., Ferstl E.C. (2012). How are affective word ratings related to lexico-semantic properties? Evidence from the Sussex Affective Word List (SAWL). Applied Psycholinguistics.

[bib12] Clark H.H. (1973). The language-as-fixed-effect fallacy: A critique of language statistics in psychological research. Journal of Verbal Learning and Verbal Behaviour.

[bib13] Colibazzi T., Posner J., Wang Z., Gorman D., Gerber A., Yu S., Russell J.A. (2010). Neural systems subserving valence and arousal during the experience of induced emotion. Emotion.

[bib14] Compton R.J., Banich M.T., Mohanty A., Milham M.P., Herrington J., Miller G.A., Heller W. (2003). Paying attention to emotion: An fMRI investigation of cognitive and emotional Stroop tasks. Cognitive, Affective & Behavioural Neuroscience.

[bib15] Craig A.D.B. (2005). Forebrain emotional asymmetry: A neuroanatomical basis?. Trends in Cognitive Sciences.

[bib16] Craig A.D.B. (2009). How do you feel now? The anterior insula and human awareness. Nature Reviews Neuroscience.

[bib17] Critchley H.D., Wiens S., Rotshtein P., Ohman A., Dolan R.J. (2004). Neural systems supporting interoceptive awareness. Nature Neuroscience.

[bib18] Dale A.M. (1999). Optimal experimental design for event-related fMRI. Human Brain Mapping.

[bib19] Damasio A.R., Grabowski T.J., Bechara A., Damasio H., Ponto L.L.B., Parvizi J., Hichwa R.D. (2000). Subcortical and cortical brain activity during the feeling of self-generated emotions. Nature Neuroscience.

[bib20] Dolcos F., LaBar K.S., Cabeza R. (2004). Dissociable effects of arousal and valence on prefrontal activity indexing emotional evaluation and subsequent memory: an event-related fMRI study. NeuroImage.

[bib21] Eder A.B., Rothermund K. (2010). Automatic influence of arousal information on evaluative processing: Valence-arousal interactions in an affective Simon task. Cognition and Emotion.

[bib22] Ellsworth P.C., Scherer K.R., Davidson R.J., Goldsmith H.H., Scherer K.R. (2003). Appraisal processes in emotion. Handbook of the affective sciences.

[bib23] Feldman Barrett L., Russell J.A. (1999). The structure of current affect: Controversies and emerging consensus. Current Directions in Psychological Science.

[bib24] Feng C., Wang L., Liu C., Zhu X., Dai R., Mai X., Luo Y.-J. (2012). The time course of the influence of valence and arousal on the implicit processing of affective pictures. PLoS ONE.

[bib25] Ferstl E.C., Neumann J., Bogler C., von Cramon D.Y. (2008). The extended language network: A meta-analysis of neuroimaging studies on text comprehension. Human Brain Mapping.

[bib26] Ferstl E.C., Rinck M., von Cramon D.Y. (2005). Emotional and temporal aspects of situation model processing during text comprehension: An event-related fMRI study. [fMRI]. Journal of Cognitive Neuroscience.

[bib27] Fiebach C.J., Friederici A.D., Mueller K., von Cramon D.Y. (2002). fMRI evidence for dual routes to the mental lexicon in visual word recognition. Journal of Cognitive Neuroscience.

[bib28] Garavan H., Pendergrass J.C., Ross T.J., Stein E.A., Risinger R.C. (2001). Amygdala response to both positively and negatively valenced stimuli. NeuroReport.

[bib29] Gray M.A., Harrison N.A., Wiens S., Critchley H.D. (2007). Modulation of emotional appraisal by false physiological feedback during fMRI. PLoS ONE.

[bib30] Hamann S., Mao H. (2002). Positive and negative emotional verbal stimuli elicit activity in the left amygdala. NeuroReport.

[bib31] Hasselmo M.E., Stern C.E. (2006). Mechanisms underlying working memory for novel information. Trends in Cognitive Sciences.

[bib32] Haxby J.V., Hoffman E.A., Gobbini M.I. (2002). Human neural systems for face recognition and social communication. Biological Psychiatry.

[bib33] Herbert C., Ethofer T., Anders S., Junghofer M., Wildgruber D., Grodd W., Kissler J. (2009). Amygdala activation during reading of emotional adjectives - an advantage for pleasant content. Social Cognitive and Affective Neuroscience.

[bib34] Hofmann M.J., Kuchinke L., Tamm S., Võ M.L.-H., Jacobs A.M. (2009). Affective processing within 1/10th of a second: High arousal is necessary for early facilitative processing of negative but not positive words. Cognitive, Affective & Behavioural Neuroscience.

[bib35] Ito T.A., Larsen J.T., Smith N.K., Cacioppo J.T. (1998). Negative information weights more heavily on the brain: The negativity bias in evaluative categorizations. Journal of Personality and Social Psychology.

[bib36] Kanske P., Kotz S.A. (2011). Emotion triggers executive attention: Anterior cingulate cortex and amygdala responses to emotional words in a conflict task. Human Brain Mapping.

[bib37] Kensinger E.A., Schacter D.L. (2006). Processing emotional pictures and words: Effects of valence and arousal. Cognitive, Affective & Behavioural Neuroscience.

[bib38] Kissler J., Assadollahi R., Herbert C. (2006). Emotional and semantic networks in visual word processing: insights from ERP studies. Progress in Brain Research.

[bib39] Kissler J., Herbert C., Peyk P., Junghofer M. (2007). Buzzwords. Early cortical responses to emotional words during reading. Psychological Science.

[bib40] Kousta S.-T., Vinson D.P., Vigliocco G. (2009). Emotion words, regardless of polarity, have a processing advantage over neutral words. Cognition.

[bib41] Kuchinke L., Jacobs A.M., Gubrich C., Võ M.L.-H., Conrad M., Herrmann M. (2005). Incidental effects of emotional valence in single word processing: An fMRI study. NeuroImage.

[bib42] Lang P.J., Bradley M.M., Cuthbert B.N., Lang P.J., Simons R.F., Balaban M.T. (1997). Motivated attention: Affect, activation, and action. Attention and orienting: Sensory and motivational processes.

[bib43] Lang P.J., Bradley M.M., Cuthbert B.N. (1999). International affective picture system (IAPS): Instruction manual and affective ratings.

[bib44] Larsen R.J., Mercer K.A., Balota D.A. (2006). Lexical characteristics of words used in emotional Stroop experiments. Emotion.

[bib45] Larsen R.J., Mercer K.A., Balota D.A., Strube M.J. (2008). Not all negative words slow down lexical decision and naming speed: Importance of word arousal. Emotion.

[bib46] Levenson R.W. (2011). Basic emotion questions. Emotion Review.

[bib47] Lewis P.A., Critchley H.D., Rotshtein P., Dolan R.J. (2007). Neural correlates of processing valence and arousal in affective words. Cerebral Cortex.

[bib48] Mathews A., MacLeod C. (1994). Cognitive approaches to emotion and emotional disorders. Annual Review of Psychology.

[bib49] *Max Planck Institute for Psycholinguistics*. Web-based CELEX. (2001). 〈http://celex.mpi.nl/〉 Retrieved 23.11.07.

[bib50] Phan K.L., Wager T., Taylor S.F., Liberzon I. (2002). Functional neuroanatomy of emotion: A meta-analysis of emotion activation studies in PET and fMRI. NeuroImage.

[bib51] Posner J., Russell J.A., Gerber A., Gorman D., Colibazzi T., Yu S., Peterson B.S. (2009). The neurophysiological bases of emotion: an fMRI study of the affective circumplex using emotion-denoting words. Human Brain Mapping.

[bib52] Price C.J. (2012). A review and synthesis of the first 20 years of PET and fMRI studies of heard speech, spoken language and reading. NeuroImage.

[bib53] Price C.J., Wise R.J.S., Frackowiak R.S.J. (1996). Demonstrating the implicit processing of visually presented words and pseudowords. Cerebral Cortex.

[bib54] Rastle K., Harrington J., Coltheart M. (2002). 358,534 nonwords: The ARC nonword database. Quarterly Journal of Experimental Psychology.

[bib55] Reisenzein R. (1994). Pleasure-arousal theory and the intensity of emotions. Journal of Personality and Social Psychology.

[bib56] Robinson M.D., Storbeck J., Meier B.P., Kirkeby B.S. (2004). Watch out! That could be dangerous: Valence-arousal interactions in evaluative processing. Personality and Social Psychology Bulletin.

[bib57] Rogers T.T., Hocking J., Noppeney U., Mechelli A., Gorno-Tempini M., Patterson K. (2006). Anterior temporal cortex and semantic memory: Reconciling findings from neuropsychology and functional imaging. Cognitive, Affective & Behavioural Neuroscience.

[bib58] Russell J.A. (2003). Core affect and the psychological construction of emotion. [Theoretical]. Psychological Review.

[bib59] Schlochtermeier L., Kuchinke L., Pehrs C., Urton K., Kappelhoff H., Jacobs A.M. (2013). Emotional picture and word processing: An fMRI study on effects of stimulus complexity. PLoS ONE.

[bib60] Small D.M., Gregory M.D., Mak Y.E., Gitelman D., Mesulam M.M., Parrish T. (2003). Dissociation of neural representation of intensity and affective valuation in human gustation. Neuron.

[bib61] Straube T., Sauer A., Miltner W.H.R. (2011). Brain activation during direct and indirect processing of positive and negative words. Behavioural Brain Research.

[bib62] Streit M., Ioannides A.A., Liu L., Wölwer W., Dammers J., Gross J., Müller-Gärtner H.-W. (1999). Neurophysiological correlates of the recognition of facial expressions of emotion as revealed by magnetoencephalography. Cognitive Brain Research.

[bib63] Ullsperger M., Harsay H.A., Wessel J.R., Ridderinkhof K.R. (2010). Conscious perception of errors and its relation to the anterior insula. Brain Structure and Function.

[bib64] Winston J.S., Gottfried J.A., Kilner J.M., Dolan R.J. (2005). Integrated neural representations of odor intensity and affective valence in human amygdala. Journal of Neuroscience.

[bib65] Wong C., Gallate J. (2012). The function of the anterior temporal lobe: A review of the empirical evidence. Brain Research.

